# Peripheral blood stem cell transplantation vs. bone marrow transplantation for aplastic anemia: a systematic review and meta-analysis

**DOI:** 10.3389/fmed.2023.1289180

**Published:** 2023-11-22

**Authors:** Zhao Zhang, Xianghui Zhou, Zhipeng Cheng, Yu Hu

**Affiliations:** Department of Hematology, Union Hospital, Tongji Medical College, Huazhong University of Science and Technology, Wuhan, China

**Keywords:** hematopoietic stem cell transplantation, peripheral blood stem cell transplantation, bone marrow transplantation, aplastic anemia, overall survival, graft-versus-host disease, transplant-related mortality, graft failure rate

## Abstract

**Background:**

Hematopoietic stem cell transplantation (HSCT) is an effective treatment for aplastic anemia. Recently, peripheral blood stem cell transplantation (PBSCT) has gradually replaced traditional bone marrow transplantation (BMT). However, which graft source has a better therapeutic effect and prognosis for aplastic anemia (AA) remains unclear. Therefore, we conducted this systematic review and meta-analysis.

**Methods:**

We systematically searched PubMed, EMBASE, and the Cochrane Library without language limitations for studies using PBSCT or BMT for AA. Data were analyzed using the Open Meta-Analyst.

**Results:**

We identified 17 of 18,749 studies, including seven comparative reports and nine single-arm reports, with a total of 3,516 patients receiving HSCT (1,328 and 2,188 patients received PBSCT and BMT, respectively). The outcomes of the comparative studies showed similar 5-year overall survival [OS; relative risk (RR) = 0.867; 95% confidence interval (CI), 0.747–1.006], similar transplant-related mortality (RR = 1.300; 95%CI, 0.790–2.138), graft failure rate (RR = 0.972; 95%CI, 0.689–1.372) between the PBSCT group and the BMT group, while the PBSCT group had a significantly higher incidence of chronic graft-versus-host disease (GVHD; RR = 1.796; 95% CI, 1.571–2.053) and a higher incidence of grade IV acute GVHD (RR = 1.560; 95% CI, 1.341–1.816) compared to the BMT group. The outcomes of single-arm reports showed similar 3-year OS and incidences of chronic GVHD, acute II–IV GVHD, III–IV GVHD, transplant-related mortality and graft failure rate between PBSCT and BMT.

**Conclusion:**

Before 2010, PBSCT was not superior to BMT in terms of 5-year OS, transplant-related mortality and graft failure rate, but it exhibited a higher risk of both chronic and acute GVHD. After 2010, PBSCT and BMT showed similar 3-year OS, GVHD risks, transplant-related mortality and graft failure rate. PB grafts are more suitable for HSCT of the AA for convenience and pain relief.

**Systematic review registration:**

www.crd.york.ac.uk/PROSPERO/, CRD42023412467.

## 1 Introduction

Aplastic anemia (AA) is a group of bone marrow hematopoietic failure syndromes caused by multiple causes, characterized by decreased proliferation of bone marrow hematopoietic cells and decreased peripheral blood whole blood cells (1). Currently, there are several new treatments for AA (2), but hematopoietic stem cell transplantation (HSCT) remains an effective treatment. Initially, all transplantations utilized bone marrow (BM) grafts for patients requiring HSCT. Some scholars still consider allogeneic bone marrow transplantation as the first-line treatment for AA (3). However, the discovery that granulocyte colony-stimulating factor (G-CSF) can collect and move cells from the bone marrow to the peripheral blood in large numbers led to peripheral blood stem cell transplantation (PBSCT) (4). With the development of technology and considering that PBSC harvesting avoids anesthesia and hospitalization and is more secure for donors (5), the choice of PBSCT for clinical treatment has gradually surpassed that of bone marrow transplantation (BMT).

However, it remains unclear whether PBSCT or BMT is better for patients with AA. Some studies have shown that the overall survival (OS) in the PBSCT group is not significantly different from that in the BMT group, and the incidence of chronic and acute graft-versus-host disease (GVHD) in the PBSCT group is significantly higher than that in the BMT group for hematologic malignancies (6–8). However, to the best of our knowledge, no high-quality studies have directly evaluated the efficacy of PBSCT vs. BMT in patients with aplastic anemia; specifically, it remains unclear whether these findings from hematologic malignancies are applicable to AA. Furthermore, previous meta-analyses on this topic have yielded controversial results.

In order to explore the efficacy of PBSCT and BMT for patients with AA, we initiated this study.

## 2 Methods

### 2.1 Search strategy and selection criteria

This study was registered with PROSPERO, a prospective international registry of systematic reviews. We conducted a search of published studies using medical subject headings and title/abstract words related to “hematopoietic stem cell transplantation,” “peripheral blood stem cell transplantation,” “bone marrow transplantation,” and “Anemia, Aplastic” in PubMed, EMBASE and the Cochrane Library without language restrictions. The detailed search strategy is described in the Supplementary material.

Studies were considered eligible if they were randomized controlled trials, prospective cohort studies, or retrospective studies that explored the efficacy of PBSCT and BMT in patients with AA. Patients with paroxysmal nocturnal hemoglobinuria, severe uncontrolled infections, or malignancies were excluded.

### 2.2 Quality assessment and data extraction

Comparisons between retrospective studies were assessed using the Newcastle-Ottawa Scale. The potential scores ranged from 0 to 10, with higher scores indicating higher quality. Single-arm studies were assessed using the Newcastle-Ottawa Scale modified by Lopez-Olivo et al. (9) for cohort studies without controls. The potential scores ranged from 0 to 6, with higher scores indicating higher quality. The following components were assessed: selection, comparability, ascertainment of exposure, and outcome.

Two authors independently extracted data. Any disagreements were resolved by discussion until a consensus was reached or by consulting a third author. If the reports were based on the same studies, the most recent report with the longest follow-up period was selected. The following data were extracted: author, year of publication, study period, inclusion criteria, total number of patients included in the study, human leukocyte antigen compatibility, conditioning regimen, OS, incidence of acute and chronic GVHD, transplant-related mortality and graft failure rate.

### 2.3 Data analysis and statistical methods

Statistical heterogeneity among the studies was evaluated using Higgins I^2^ statistics. If I^2^ was 50%, the data were combined using a random effects model. Otherwise, a fixed effects model was used. We assessed the outcomes using the relative risk (RR) with a 95% confidence interval (CI) for retrospective reports, directly comparing the treatment outcomes of BMT and PBSCT. If 95% CIs of outcomes excluded “1,” we concluded that the outcomes were statistically significant. Moreover, we conducted separate analyses of single-arm retrospective studies on PBSCT and BMT treatment outcomes. Dichotomous data obtained from each study were expressed as proportions. If the 95% CIs of outcomes among the different graft source groups did not overlap, we concluded that the outcomes were statistically significant. All studies were analyzed using the Open Meta-Analyst software.

## 3 Results

### 3.1 Search results and study characteristics

**Figure 1:**
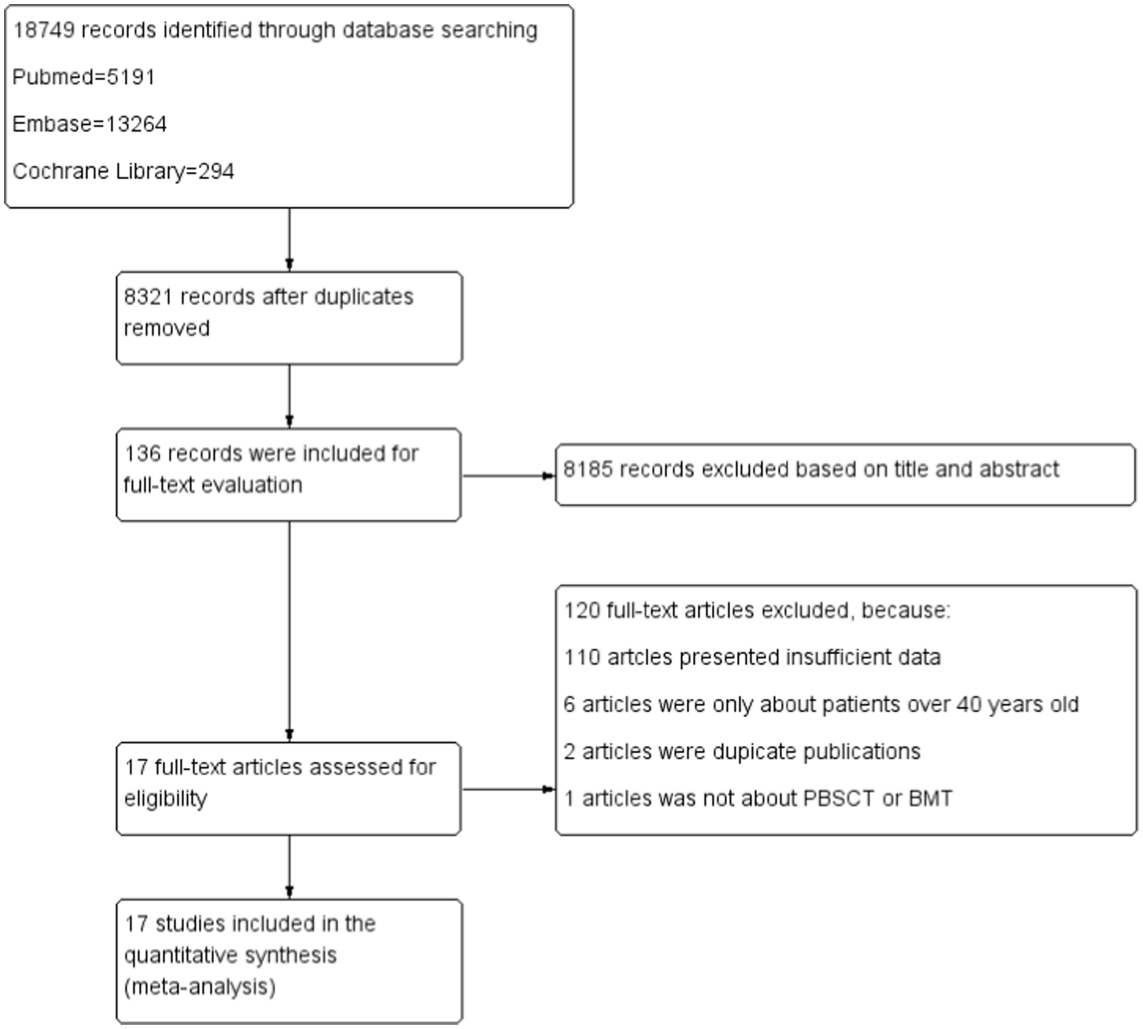
Flow diagram of the study selection procedure.

We initially identified 18,749 studies in PubMed, EMBASE, and the Cochrane Library, of which 8,321 duplicate studies were removed. After screening the titles and abstracts, we excluded 8,185 studies. A careful review of the full texts indicated that 17 studies were eligible, including seven retrospective reports that directly compared the treatment outcomes of BMT and PBSCT and 10 single-arm studies that only examined the treatment outcomes of PBSCT or BMT. The detailed process of study selection and identification is shown in Figure 1.

The general characteristics of the studies and patients are summarized in Table 1, including the author, year of publication, total number of patients included in the study, type of study, median age of patients, GVHD prophylaxis, and NOS scores of the studies.

**Table 1:** Characteristics of studies included.

Study	Study arm	Number of patients	Study design	Median age	GVHD prophylaxis	Donor types	NOS score
Wang et al. (10)	PB	23	Retrospective	NA	CsA + MTX CsA + MTX+ MMF	HLA-matched sibling donor and unrelated donor	6
	BM	10		NA			
Ghavamzadeh et al. (11)	PB	145	Retrospective	24 (2–50)	CsA + MTX + Cy CsA + MTX + Cy + ATG	HLA-matched sibling donor	7
	BM	40		17.5 (1–32)			
Chen et al. (12)	PB	24	Retrospective	NA	CsA + MTX TAC + MTX	HLA-matched sibling donor	6
	BM	17		NA			
Schrezenmeier et al. (13)	PB	134	Retrospective	NA	CsA ± other CsA + MTX ± other TAC ± other	HLA-matched sibling donor	6
	BM	558		NA			
Eapen et al. (14)	PB	71	Prospective	36 (1–71)	CsA + MTX ± other CsA ± other TAC MMF + other TAC + other	HLA-matched unrelated donor	8
	BM	225		19 (2–66)			
Bacigalupo et al. (15)	PB	723	Prospective	24 (1–69)	CsA CsA ± other CsA + MTX CsA + MTX ± other	HLA-matched sibling donor	7
	BM	1,163		18 (1–68)			
George et al. (16)	PB	7	Retrospective	7 (5–15)	CsA + MTX	HLA-matched sibling donor	6
	BM	7		12 (6–13)			
Aladag et al. (17)	PB	27	Retrospective	24 (17–55)	CsA + MTX TAC + MTX	HLA-matched sibling donor	6
Shu-Qing et al. (18)	PB	26	Prospective	NA	NA	Haploidentical donor	5
Suping et al. (19)	PB	21	Retrospective	NA	CsA + MMF + MTX	HLA-matched unrelated donor	5
Davulcu et al. (20)	PB	13	Retrospective	NA	CsA+ MTX	HLA-matched sibling donor and unrelated donor	5
Liang (21)	PB	22	Prospective	NA	CsA + Cy + ATG	Haploidentical donor	6
Nakamura et al. (22)	PB	94	Retrospective	39 (16–68)	CsA TAC ATG + CsA ATG + TAC	HLA-matched sibling donor	7
Liu et al. (23)	BM	22	Prospective	17 (5–35)	TAC + MMF + MTX	Haploidentical donor	6
Kako et al. (24)	BM	26	Prospective	36 (18–61)	CsA + MTX TAC + MTX	HLA-matched or one locus-mismatched related donor, or an HLA-matched or one DRB1 allele-mismatched unrelated donor	6
Lu et al. (25)	BM	89	Retrospective	NA	NA	Haploidentical donor and HLA-matched unrelated donor	5
DeZern et al. (26)	BM	31	Prospective	NA	Cy + ATG + Flu + TAC + MMF	Haploidentical donor	5

CsA, cyclosporin A; Cy, cyclophosphamide; MTX, methotrexate; TAC, tacrolimus; ATG, antithymocyte globulin; MMF, mycophenolate mofetil; Flu, fludarabine; NA, not available.

### 3.2 Overall survival

**Figure 2:**
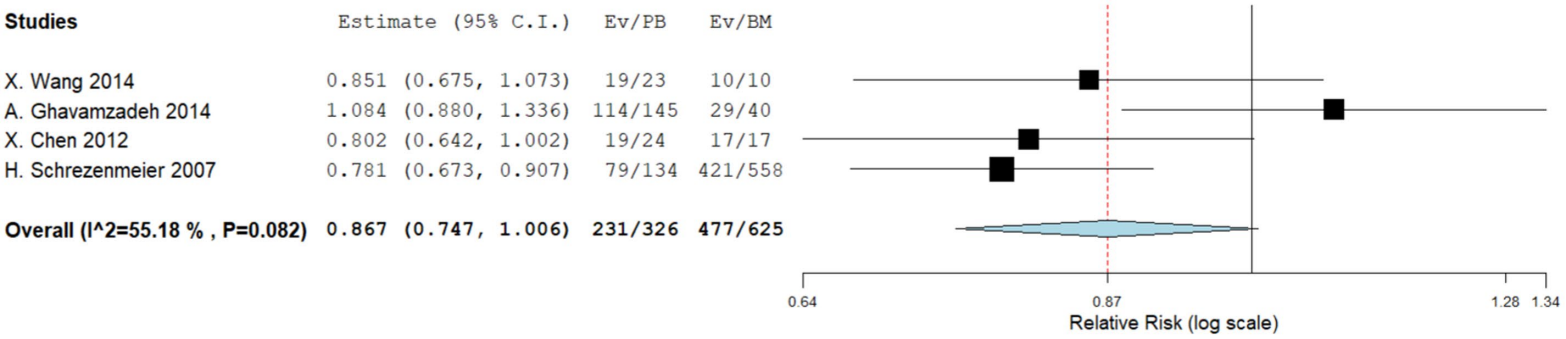
Five-year overall survival in comparing studies. A forest plot illustration. CI, confidence interval.

**Figure 3:**
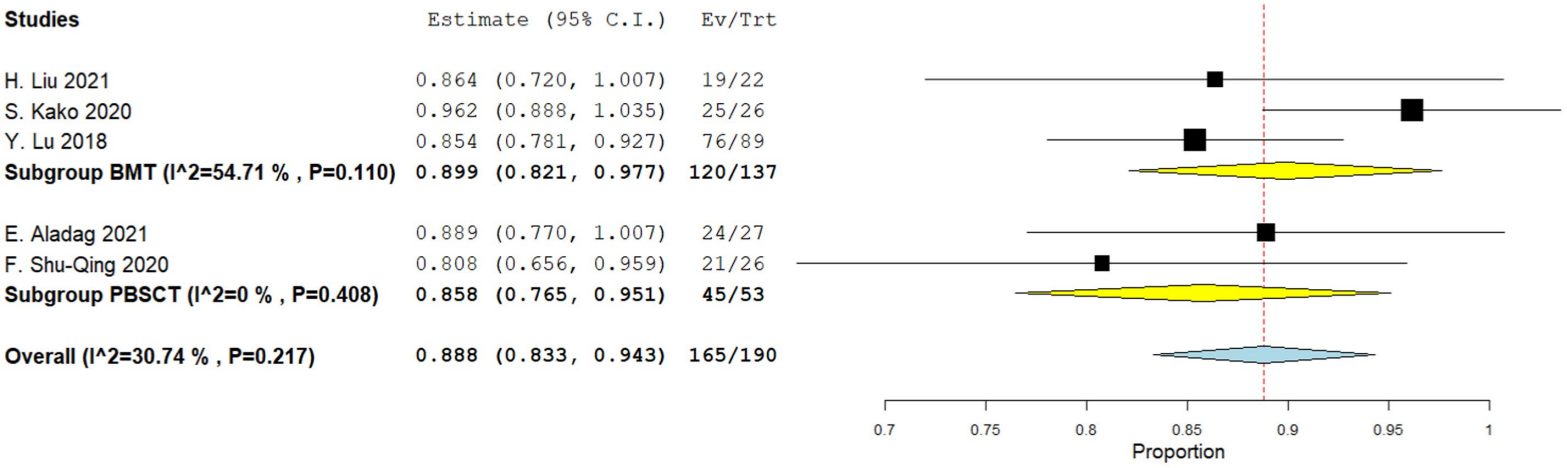
Three-year overall survival in single-arm studies. A forest plot illustration. CI, confidence interval; BMT, bone marrow transplantation; PBSCT, peripheral blood stem cell transplantation.

Four comparative studies reported data on the 5-year OS. No significant difference was observed in the 5-year OS between the two groups. The RR for the 5-year OS of the comparative studies was 0.867 (95%CI: 0.747–1.006, I^2^ = 55.18%, Figure 2). Five single-arm studies reported 3-year OS rates. There was no significant difference in the 3-year OS between the two groups. The 3-year OS rates of the single-arm PBSCT and BMT studies were 0.858 (95%CI, 0.765–0.951, I^2^ = 0%; Figure 3) and 0.899 (95%CI, 0.821–0.977, I^2^ = 54.71%, Figure 3), respectively.

### 3.3 Chronic GVHD

**Figure 4:**
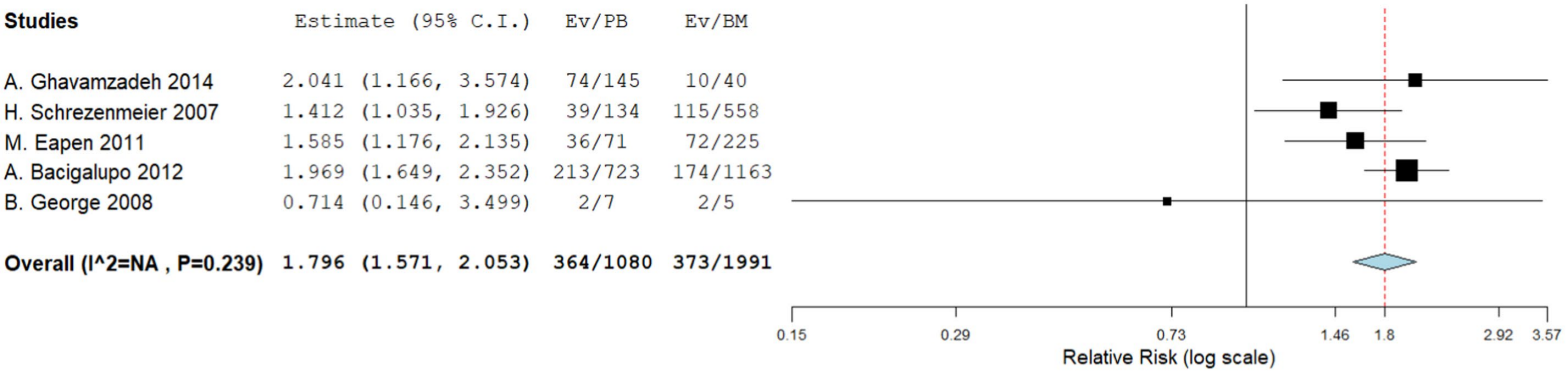
Chronic GVHD in comparing studies. A forest plot illustration. CI, confidence interval.

**Figure 5:**
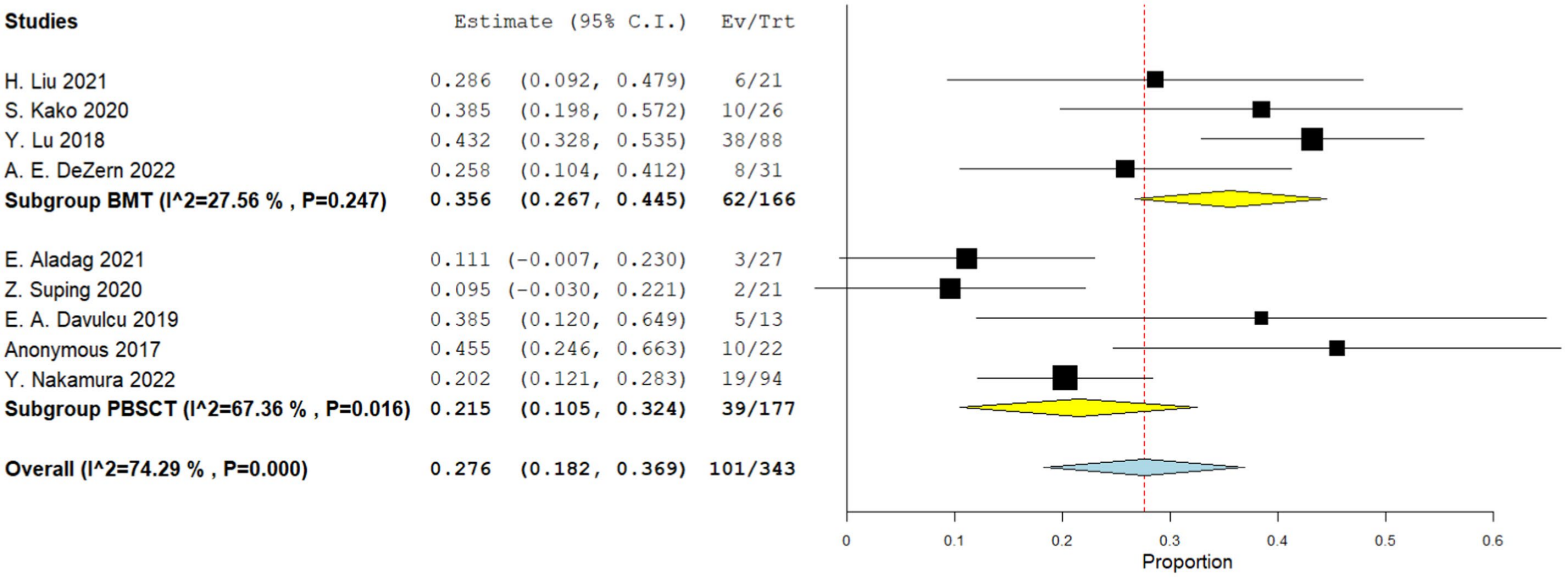
Chronic GVHD in single arm studies. A forest plot illustration. CI, Confidence Interval; BMT, bone marrow transplantation; PBSCT, peripheral blood stem cell transplantation.

Five comparative studies focused on chronic GVHD. These studies revealed a significantly higher incidence of chronic GVHD in the PBSCT group. The RR for chronic GVHD in the comparative studies was 1.796 (95%CI, 1.571–2.053, I^2^ = 26.616%, Figure 4). Nine single-arm studies reported chronic GVHD. No significant difference in the incidence of chronic GVHD was observed between the two groups. The incidence of chronic GVHD in the single-arm PBSCT and BMT studies was 0.215 (95%CI, 0.105–0.324, I^2^ = 67.36%; Figure 5) and 0.356 (95%CI, 0.267–0.445, I^2^ = 27.56%; Figure 5), respectively.

### 3.4 Acute GVHD

**Figure 6:**
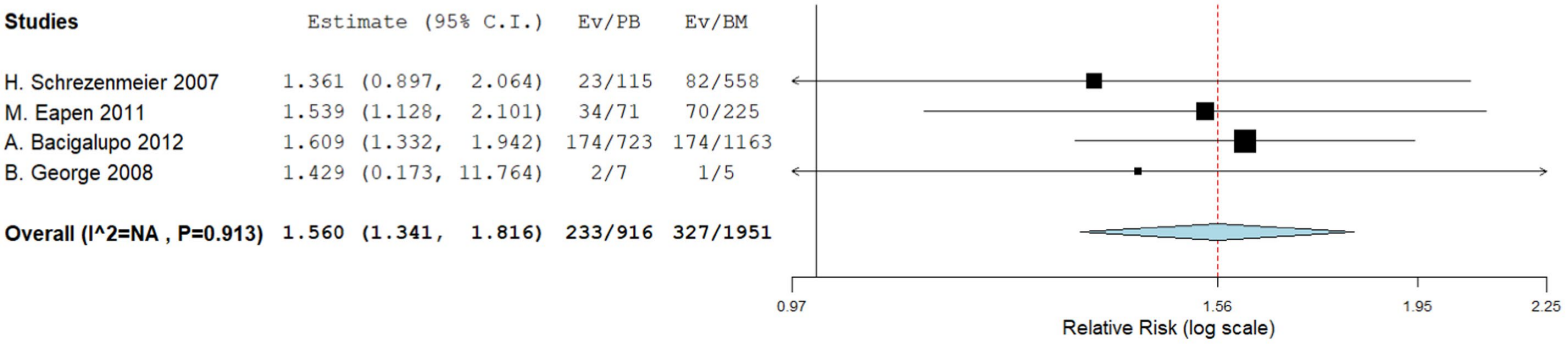
Grade II–IV acute GVHD in comparing studies. A forest plot illustration. CI, Confidence Interval.

**Figure 7:**
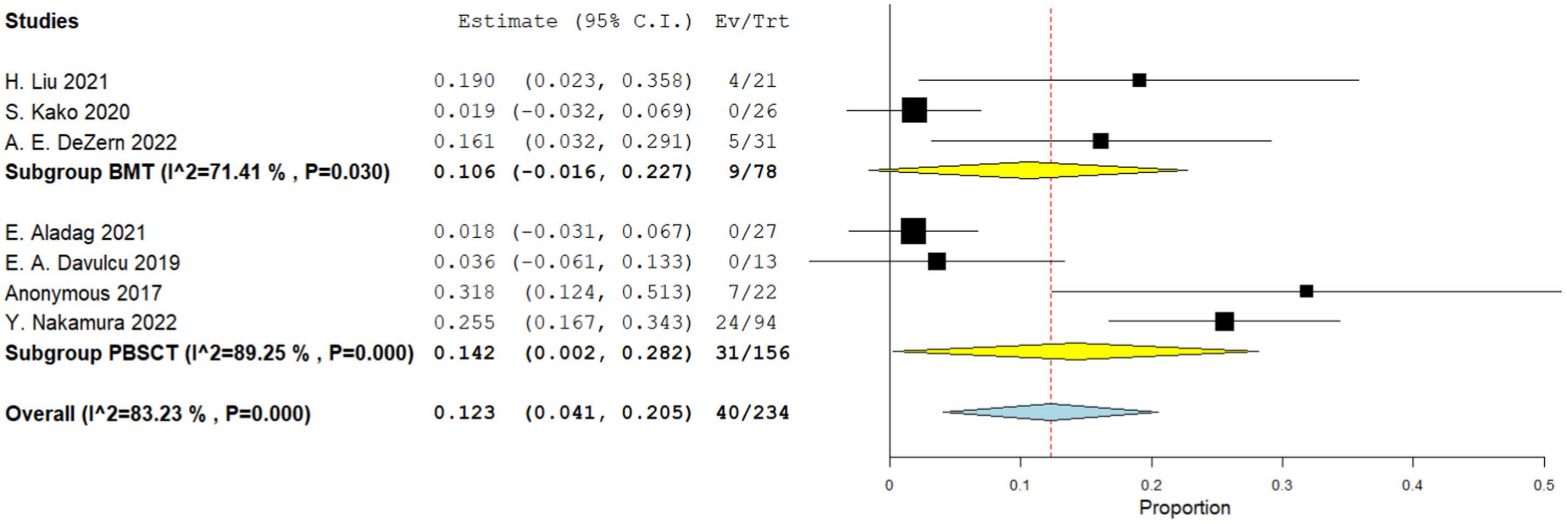
Grade II–IV acute GVHD in single arm studies. A forest plot illustration. CI, Confidence interval; BMT, bone marrow transplantation; PBSCT, peripheral blood stem cell transplantation.

**Figure 8:**
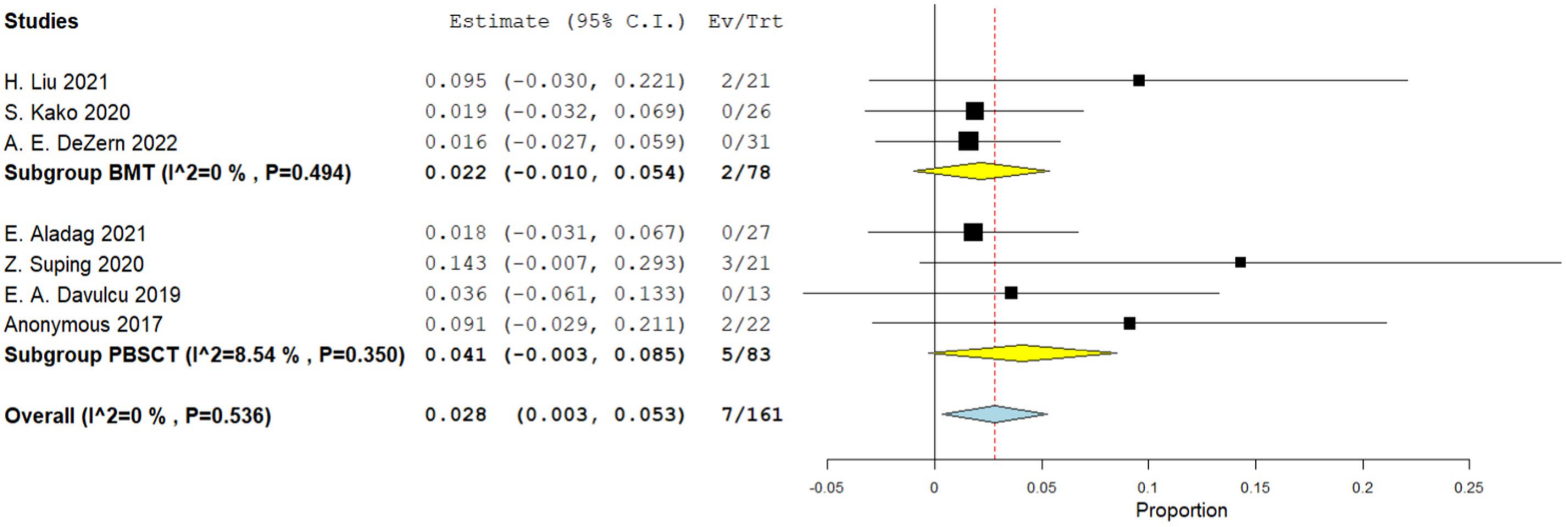
Grade II–IV acute GVHD in single arm studies. A forest plot illustration. CI, Confidence interval; BMT, bone marrow transplantation; PBSCT, peripheral blood stem cell transplantation.

Four comparative studies reported on grade IV acute GVHD, showing that patients who underwent PBSCT had a higher risk of grade IV acute GVHD. The RR for II–IV acute GVHD in the comparative studies was 1.560 (95%CI, 1.341–1.816, I^2^ = 0%, Figure 6). Seven single-arm studies reported on stage II–IV acute GVHD. No difference in the incidence of grade IV acute GVHD was observed between the two groups. The incidence of II–IV acute GVHD of the single arm PBSCT and BMT studies were 0.142 (95%CI, 0.002–0.282, I^2^ = 89.25%, Figure 7) and 0.106 (95%CI, −0.016–0.227, I^2^ = 71.41%, Figure 7), respectively. Seven single-arm studies reported stage III–IV acute GVHD. No significant differences were found between the two groups. The incidences of III–IV acute GVHD in the single-arm PBSCT and BMT studies were 0.041 (95%CI, −0.003–0.085, I^2^ = 8.54%, Figure 8) and 0.022 (95%CI, −0.010–0.054, I^2^ = 0%, Figure 8), respectively.

### 3.5 Transplant-related mortality

**Figure 9:**
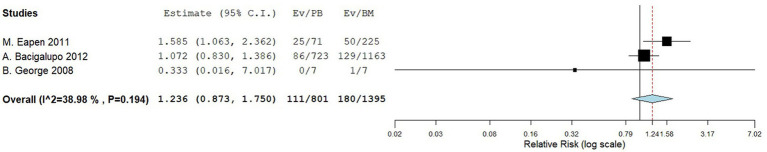
Transplant-related mortality in comparing studies. A forest plot illustration. CI, confidence interval.

**Figure 10:**
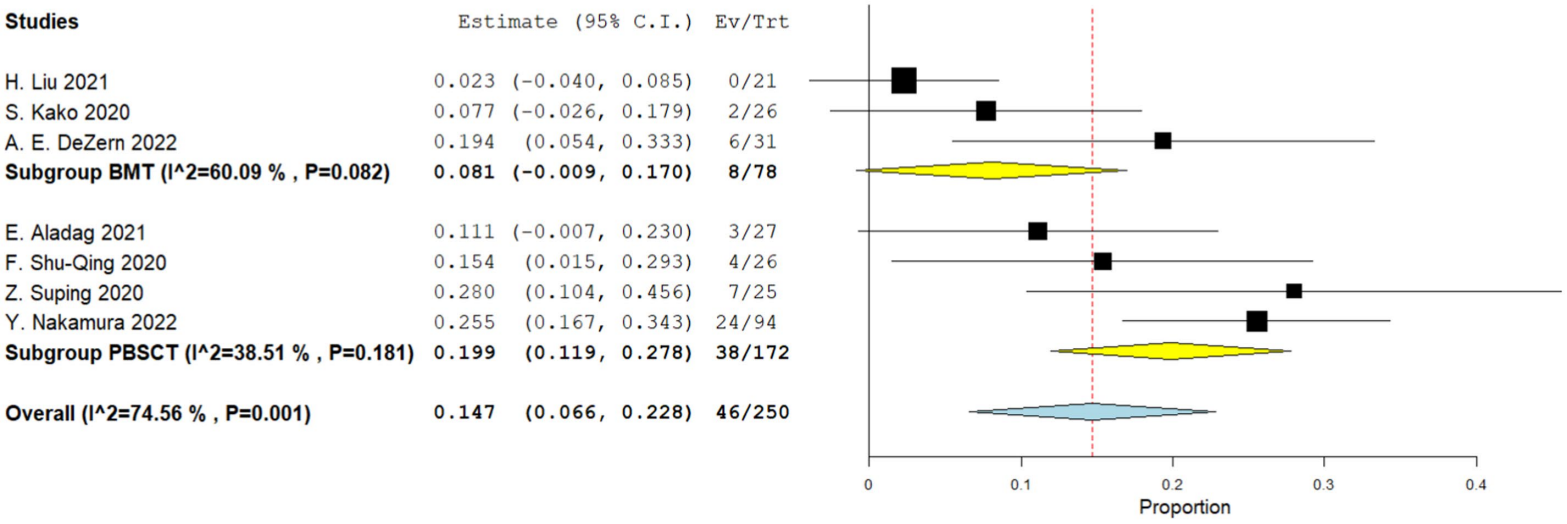
Transplant-related mortality in single-arm studies. A forest plot illustration. CI, Confidence Interval; BMT, bone marrow transplantation; PBSCT, peripheral blood stem cell transplantation.

Three comparative studies reported data on the number of transplant related death. No significant difference was observed in the transplant-related mortality between the two groups. The RR for the transplant-related mortality of the comparative studies was 1.236 (95%CI: 0.873–1.750, I^2^ = 38.98%, Figure 9). Seven single-arm studies reported the number of transplant related death. There was no significant difference in the transplant-related mortality between the two groups. The transplant-related mortality of the single-arm PBSCT and BMT studies were 0.199 (95%CI, 0.119–0.278, I^2^ = 38.51%; Figure 10) and 0.081 (95%CI, −0.009–0.170, I^2^ = 60.09%; Figure 10), respectively.

### 3.6 Graft failure rate

**Figure 11:**
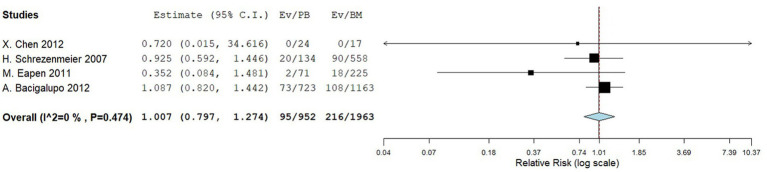
Graft failure rate in comparing studies. A forest plot illustration. CI, confidence interval.

**Figure 12:**
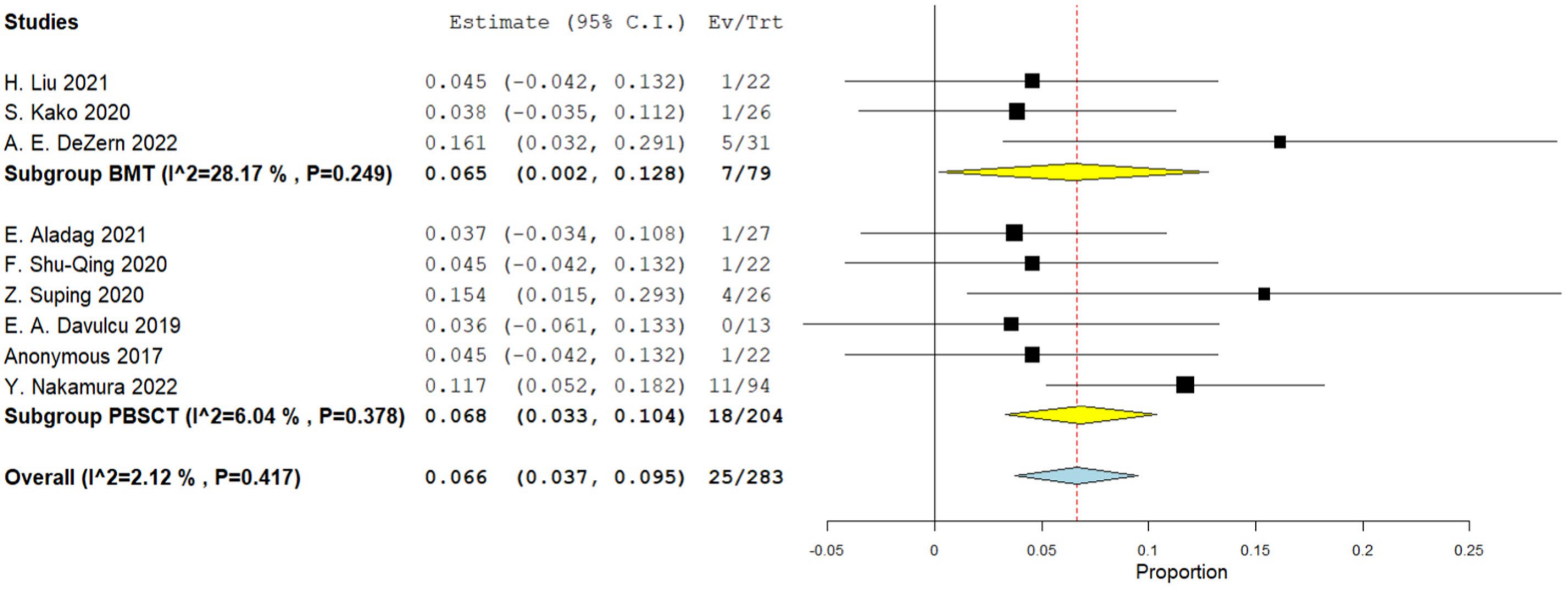
Graft failure rate in single-arm studies. A forest plot illustration. CI, Confidence Interval; BMT, bone marrow transplantation; PBSCT, peripheral blood stem cell transplantation.

Four comparative studies reported data on the number of graft failure. There is no significant difference in the graft failure rate between the two groups. The RR for the graft failure rate of the comparative studies was 1.007 (95%CI: 0.797–1.274, I^2^ = 0%, Figure 11). Nine single-arm studies reported graft failure rate. No significant difference was observed in the graft failure rate between the two groups. The graft failure rate of the single-arm PBSCT and BMT studies were 0.068 (95%CI, 0.033–0.104, I^2^ = 6.04%; Figure 12) and 0.065 (95%CI, 0.002–0.128, I^2^ = 28.17%; Figure 12), respectively.

## 4 Discussion

Some studies have reported differences between PBSCT and BMT in malignant hematological diseases; however, there are very few related reports on non-malignant hematological diseases. To the best of our knowledge, this is the first meta-analysis to report a difference in efficacy between PBSCT and BMT in AA. This review included both comparative and single-arm studies. In some single-arm studies involving subgroup comparisons, such as Lu′s study (25), subgroups were merged into the study if no statistically significant difference existed between them; otherwise, the data were excluded.

Comparative studies have shown that PBSCT and BMT groups have similar 5-year OS rates, transplant-related mortality and graft failure rate. The single-arm study provided similar results, with no statistical differences in the 3-year survival rates, transplant-related mortality and graft failure rate between the PBSCT and BMT groups. In comparing acute and chronic GVHD between the two groups, we found that the incidence of GVHD in patients with AA in the PBSCT group was significantly higher than that in the BMT group, regardless of whether they had acute or chronic GVHD. However, this phenomenon was not observed in single-arm studies in which the two groups had similar acute and chronic GVHD incidences. As is well known, the number of T cells in PB is 10 times that of BM. High concentrations of CD34+ T cells in the PB are likely to induce GVHD (27). This aligns with the results of comparative studies presented in this research and is also consistent with findings from studies examining differences between PBSCT and BMT in malignant hematological diseases. However, in the single-arm studies, both groups showed a similar incidence of GVHD. Through comparison, we found that the included comparative studies were published around 2010, whereas the single-arm studies were published around 2020. Therefore, we reviewed the relevant diagnostic and treatment guidelines and expert consensus published between 2010 and 2020. The 2009 UK guidelines did not recommend PB as a graft source for patients with AA (28) and the expert consensus from China in 2010 mentioned only BMT as the recommended treatment plan for patients (29). In the 2020 Hematopoietic Cell Guidelines from the NCCN (30), the task force did not differentiate recommendations for allogeneic HSCT indications based on graft source but emphasized the necessity of researching the efficacy of different graft sources. Similarly, the 2022 Chinese guidelines for the diagnosis and treatment of AA only mention the use of HSCT for the treatment of AA without discussing the source of the graft (31). To some extent, these guidelines and consensus conclusions are consistent with the conclusions of this study. We believe that patient acceptance of PBSCT is increasing and that, with the maturation of both PBSCT and GVHD prophylaxis, PBSCT no longer presents the same high risk of GVHD for patients with AA as it did in the past.

Our meta-analysis has some limitations. First, the outcomes reported in the included studies may differ; therefore, it is not possible to gather sufficient and necessary data, such as disease-free survival and engraftment rate, to comprehensively compare the efficacy of PBSCT and BMT for patients with AA. Second, the sample size of individual studies in our meta-analysis was small, which resulted in a wide range of 95% CIs. Third, owing to the different design ideas, baselines, and patient sources of each study, there were differences in the age composition and treatment plans of each study, which may have interfered with the outcomes of our study. We hope to use a larger sample size and report as much data as possible to enrich this study.

Here, we evaluated the efficacy of PBSCT and BMT in patients with AA. Our study suggests that PBSCT and BMT have similar OS rates, transplant-related mortality, graft failure rate, and the incidence of GVHD after PBSCT is not higher than that after BMT. Considering its economic benefits, convenience for doctors, and donor safety, PBSCT appears to be the better choice.

## Data availability statement

The original contributions presented in the study are included in the article/Supplementary material, further inquiries can be directed to the corresponding authors.

## Author contributions

ZZ: Data curation, Formal Analysis, Investigation, Methodology, Software, Visualization, Writing – original draft, Writing – review & editing. XZ: Data curation, Formal Analysis, Investigation, Methodology, Software, Visualization, Writing – original draft, Writing – review & editing. ZC: Conceptualization, Funding acquisition, Project administration, Validation, Writing – review & editing. YH: Conceptualization, Funding acquisition, Project administration, Validation, Writing – review & editing.

## References

[1] Javan MR Saki N Moghimian-Boroujeni B. Aplastic anemia, cellular and molecular aspects. Cell Biol Int. (2021) 45:2395–402. doi: 10.1002/cbin.11689, PMID: 34405925

[2] Fattizzo B Motta I. Rise of the planet of rare anemias: an update on emerging treatment strategies. Front Med. (2022) 9:1097426. doi: 10.3389/fmed.2022.1097426 PMC9868867 36698833

[3] Georges GE Doney K Storb R. Severe aplastic anemia: allogeneic bone marrow transplantation as first-line treatment. Blood Adv. (2018) 2:2020–8. doi: 10.1182/bloodadvances.2018021162 30108110 PMC6093726

[4] Dreger P Haferlach T Eckstein V Jacobs S Suttorp M Löuffler H . G-Csf-mobilized peripheral blood progenitor cells for allogeneic transplantation: safety, kinetics of mobilization, and composition of the graft. Br J Haematol. (1994) 87:609–13. doi: 10.1111/j.1365-2141.1994.tb08321.x 7527648

[5] Pulsipher MA Chitphakdithai P Miller JP Logan BR King RJ Rizzo JD . Adverse events among 2408 unrelated donors of peripheral blood stem cells: results of a prospective trial from the National Marrow Donor Program. Blood. (2009) 113:3604–11. doi: 10.1182/blood-2008-08-175323 19190248 PMC2668845

[6] Zhang H Chen J Que W. Allogeneic peripheral blood stem cell and bone marrow transplantation for hematologic malignancies: meta-analysis of randomized controlled trials. Leuk Res. (2012) 36:431–7. doi: 10.1016/j.leukres.2011.10.016, PMID: 22050904

[7] Yu X Liu L Xie Z Dong C Zhao L Zhang J . Bone marrow versus peripheral blood as a graft source for haploidentical donor transplantation in adults using post-transplant cyclophosphamide-a systematic review and meta-analysis. Crit Rev Oncol Hematol. (2019) 133:120–8. doi: 10.1016/j.critrevonc.2018.05.017, PMID: 30661648

[8] Holtick U Albrecht M Chemnitz JM Theurich S Shimabukuro-Vornhagen A Skoetz N . Comparison of bone marrow versus peripheral blood allogeneic hematopoietic stem cell transplantation for hematological malignancies in adults—a systematic review and meta-analysis. Crit Rev Oncol Hematol. (2015) 94:179–88. doi: 10.1016/j.critrevonc.2014.12.007, PMID: 25604498

[9] Lopez-Olivo MA Pratt G Palla SL Salahudeen A. Rasburicase in tumor lysis syndrome of the adult: a systematic review and meta-analysis. Am J Kidney Dis. (2013) 62:481–92. doi: 10.1053/j.ajkd.2013.02.378 23684124

[10] Wang X Jin S Liu L Chen S Zhang X Jin Z . Efficacies of hematopoietic stem cell transplantation for severe aplastic anemia: a report of 43 patients. Zhonghua Yi Xue Za Zhi. (2014) 94:3140–4. doi: 10.3760/cma.j.issn.0376-2491.2014.40.006 25573308

[11] Ghavamzadeh A Hamidieh A Alimoghaddm K Aliabadi L Jalali A Jalili M. A comparison between peripheral blood stem cell transplantation and bone marrow transplantation as progenitor cell source in severe aplastic anemia. Bone Marrow Transplant. (2014) 20:S110. doi: 10.1016/j.bbmt.2013.12.157

[12] Chen X Wei JL Huang Y He Y Yang DL Jiang EL . Outcome of allogeneic hematopoietic stem cell transplantation from Hla-matched sibling donor for 41 cases of severe aplastic anemia. Zhonghua Xue Ye Xue Za Zhi. (2012) 33:610–4. doi: 10.3760/cma.j.issn.0253-2727.2012.08.005 PMID: 23134851

[13] Schrezenmeier H Passweg JR Marsh JCW Bacigalupo A Bredeson CN Bullorsky E . Worse outcome and more chronic Gvhd with peripheral blood progenitor cells than bone marrow in Hla-matched sibling donor transplants for young patients with severe acquired aplastic anemia. Blood. (2007) 110:1397–400. doi: 10.1182/blood-2007-03-081596, PMID: 17475907 PMC1939910

[14] Eapen M Le Rademacher J Antin JH Champlin RE Carreras J Fay J . Effect of stem cell source on outcomes after unrelated donor transplantation in severe aplastic anemia. Blood. (2011) 118:2618–21. doi: 10.1182/blood-2011-05-354001, PMID: 21677312 PMC3167362

[15] Bacigalupo A Socié G Schrezenmeier H Tichelli A Locasciulli A Fuehrer M . Bone marrow versus peripheral blood as the stem cell source for sibling transplants in acquired aplastic anemia: survival advantage for bone marrow in all age groups. Haematologica. (2012) 97:1142–8. doi: 10.3324/haematol.2011.054841, PMID: 22315497 PMC3409810

[16] George B Mathews V Viswabandya A Kavitha ML Srivastava A Chandy M. Fludarabine based reduced intensity conditioning regimens in children undergoing allogeneic stem cell transplantation for severe aplastic anemia. Pediatr Transplant. (2008) 12:14–9. doi: 10.1111/j.1399-3046.2007.00825.x, PMID: 18086256

[17] Aladag E Goker H Demiroglu H Aksu S Sayinalp N Haznedaroglu IC . Long-term results of allogeneic peripheral blood hematopoietic stem cell transplantation for severe aplastic anemia. Transfus Apher Sci. (2021) 60:103050. doi: 10.1016/j.transci.2020.103050, PMID: 33446450

[18] Shu-Qing F Hui X Cui-Min W Yan-Hong Y Yue S Zhi-Bin L . Optimization and clinical application of the preconditioning regimen in the treatment of severe aplastic anemia with interfamilial haploidentical peripheral hematopoietic stem cell transplantation: a report of 26 cases. Med J ChinPeoples Liberat Army. (2020) 45:962–6. doi: 10.11855/j.issn.0577-7402.2020.09.10

[19] Suping Z Ling S Dingming W Weijie C Li L Changfeng L . Effectiveness of unrelated peripheral blood stem cell transplantation in the treatment of severe aplastic anemia. Chin J Tissue Engineer Res. (2020) 24:4994–5001. doi: 10.3969/j.issn.2095-4344.2130

[20] Davulcu EA Bülbül H Ulusoy Y Neupane K Rafae A Mahmood SK . Allogeneic hematopoietic stem cell transplantation in adult aplastic anaemia patients: promising treatment modality. Bone Marrow Transplant. (2019) 54:173–4. doi: 10.1038/s41409-019-0559-4 30108324

[21] Liang SY. Improved outcome of haploidentical peripheral blood stem cell transplant treating severe aplastic anemia. Blood. (2017) 130:3227. doi: 10.1182/blood.V130.Suppl_1.3227.3227

[22] Nakamura Y Mori T Kako S Yamazaki H Kanda Y Uchida N . Outcome of peripheral blood stem cell transplantation from Hla-identical sibling donors for adult patients with aplastic anemia. Int J Hematol. (2022) 117:356–65. doi: 10.1007/s12185-022-03487-6 36378405

[23] Liu H Zheng X Zhang C Xie J Gao B Shao J . Outcomes of haploidentical bone marrow transplantation in patients with severe aplastic anemia-ii that progressed from non-severe acquired aplastic anemia. Front Med. (2021) 15:718–27. doi: 10.1007/s11684-020-0807-4, PMID: 34170455

[24] Kako S Kanda Y Onizuka M Aotsuka N Usuki K Tachibana T . Allogeneic hematopoietic stem cell transplantation for aplastic anemia with pre-transplant conditioning using fludarabine, reduced-dose cyclophosphamide, and low-dose thymoglobulin: a Ksgct prospective study. Am J Hematol. (2020) 95:251–7. doi: 10.1002/ajh.25693 31804748

[25] Lu Y Sun RJ Zhao YL Xiong M Cao XY Zhang JP . Unmanipulated haploidentical hematopoietic stem cell transplantation achieved outcomes comparable with matched unrelated donor transplantation in young acquired severe aplastic Anemia. Biol Blood Marrow Transplant. (2018) 24:1881–7. doi: 10.1016/j.bbmt.2018.05.015, PMID: 29772350

[26] Dezern AE Eapen M Wu J Talano JA Solh M Dávila Saldaña BJ . Haploidentical bone marrow transplantation in patients with relapsed or refractory severe aplastic anaemia in the Usa (Bmt Ctn 1502): a multicentre, single-arm, phase 2 trial. Lancet Haematol. (2022) 9:e660–9. doi: 10.1016/S2352-3026(22)00206-X, PMID: 35907408 PMC9444987

[27] Cao TM Shizuru JA Wong RM Sheehan K Laport GG Stockerl-Goldstein KE . Engraftment and survival following reduced-intensity allogeneic peripheral blood hematopoietic cell transplantation is affected by Cd8+ T-cell dose. Blood. (2005) 105:2300–6. doi: 10.1182/blood-2004-04-1473, PMID: 15572597

[28] Marsh JC Ball SE Cavenagh J Dokal I Gordon-Smith EC Keidan J . Guidelines for the diagnosis and management of aplastic anaemia. Br J Haematol. (2009) 147:43–70. doi: 10.1111/j.1365-2141.2009.07842.x 19673883

[29] Zhang FK Zhang L Zheng YZ. Several discussions about expert consensus on diagnosis and treatment of aplastic anemia. Zhonghua Xue Ye Xue Za Zhi. (2011) 32:359–60. doi: 10.3760/cma.j.issn.0253-2727.2011.05.020 21729613

[30] Kanate AS Majhail NS Savani BN Bredeson C Champlin RE Crawford S . Indications for hematopoietic cell transplantation and immune effector cell therapy: guidelines from the American Society for Transplantation and Cellular Therapy. Biol Blood Marrow Transplant. (2020) 26:1247–56. doi: 10.1016/j.bbmt.2020.03.002, PMID: 32165328

[31] Red Blood Cell Disease (Anemia) Group, Chinese Society of Hematology, Chinese Medical Association. Guidelines for the diagnosis and management of aplastic anemia in China. Zhonghua Xue Ye Xue Za Zhi. (2022) 43:881–8. doi: 10.3760/cma.j.issn.0253-2727.2022.11.001, PMID: 36709177 PMC9808872

