# Supplementary material for: Peripheral blood stem cell transplantation vs. bone marrow transplantation for aplastic anemia: a systematic review and meta-analysis
Source: Front Med (Lausanne). 2023 Nov 22;10:1289180. doi: 10.3389/fmed.2023.1289180 (PMC10702595; doi:10.3389/fmed.2023.1289180)
Supplement: Supplementary file 1 [file Data_Sheet_1.docx]

**PUBMED (5,191)**

#1. ((("Hematopoietic Stem Cell Transplantation"[Mesh]) OR (Hematopoietic Stem Cell Transplantation[Title/Abstract])) OR (Stem Cell Transplantation, Hematopoietic[Title/Abstract])) OR (Transplantation, Hematopoietic Stem Cell[Title/Abstract]) (63,128)

#2. (((("Peripheral Blood Stem Cell Transplantation"[Mesh]) OR (Peripheral Blood Stem Cell Transplantation[Title/Abstract])) OR (Peripheral Stem Cell Transplantation[Title/Abstract])) OR (Stem Cell Transplantation, Peripheral[Title/Abstract])) OR (Transplantation, Peripheral Stem Cell[Title/Abstract]) (5,913)

#3. (((((("Bone Marrow Transplantation"[Mesh]) OR (Bone Marrow Transplantation[Title/Abstract])) OR (Grafting, Bone Marrow[Title/Abstract])) OR (Bone Marrow Grafting[Title/Abstract])) OR (Transplantation, Bone Marrow[Title/Abstract])) OR (Bone Marrow Cell Transplantation[Title/Abstract])) OR (Transplantation, Bone Marrow Cell[Title/Abstract]) (54,615)

#4. ((((((((("Anemia, Aplastic"[Mesh]) OR (Anemia, Aplastic[Title/Abstract])) OR (Aplastic Anemias[Title/Abstract])) OR (Aplastic Anemia[Title/Abstract])) OR (Aplastic Anaemia[Title/Abstract])) OR (Anaemia, Aplastic[Title/Abstract])) OR (Aplastic Anaemias[Title/Abstract])) OR (Anemia, Hypoplastic[Title/Abstract])) OR (Hypoplastic Anemia[Title/Abstract])) OR (Hypoplastic Anemias[Title/Abstract]) (20,745)

#5. #1 AND #4 (1,762)

#6. #2 AND #4 (189)

#7. #3 AND #4 (3,240)

**Embase (13,264)**

#1. 'hematopoietic stem cell transplantation'/exp OR 'hematopoietic stem cell transplantation' (90719)

#2. 'hematopoietic stem cell transplantation':ab,ti OR 'stem cell transplantation, hematopoietic':ab,ti OR 'transplantation, hematopoietic stem cell':ab,ti (41,094)

#3. 'peripheral blood stem cell transplantation'/exp OR 'peripheral blood stem cell transplantation' (8,719)

#4. 'peripheral blood stem cell transplantation':ab,ti OR 'peripheral stem cell transplantation':ab,ti OR 'stem cell transplantation, peripheral':ab,ti OR 'transplantation, peripheral stem cell':ab,ti (4,529)

#5. 'bone marrow transplantation'/exp OR 'bone marrow transplantation' (112,229)

#6. 'bone marrow transplantation':ab,ti OR 'grafting, bone marrow':ab,ti OR 'bone marrow grafting':ab,ti OR 'transplantation, bone marrow':ab,ti OR 'bone marrow cell transplantation':ab,ti OR 'transplantation, bone marrow cell':ab,ti (35,437)

#7. 'aplastic anemia'/exp OR 'aplastic anemia' (53,959)

#8. 'anemia, aplastic':ab,ti OR 'aplastic anemias':ab,ti OR 'aplastic anemia':ab,ti OR 'aplastic anaemia':ab,ti OR 'anaemia, aplastic':ab,ti OR 'aplastic anaemias':ab,ti OR 'anemia, hypoplastic':ab,ti OR 'hypoplastic anemia':ab,ti OR 'hypoplastic anemias':ab,ti (16,671)

#9. (#1 or #2) and (#7 or #8) (5,442)

#10. (#3 or #4) and (#7 or #8) (588)

#11. (#5 or #6) and (#7 or #8) (7,234)

**Cochrane Library (294)**

#1. MeSH descriptor: [Hematopoietic Stem Cell Transplantation] explode all trees (1,956)

#2. (Hematopoietic Stem Cell Transplantation or Stem Cell Transplantation, Hematopoietic or Transplantation, Hematopoietic Stem Cell):ti,ab,kw (4,951)

#3. MeSH descriptor: [Peripheral Blood Stem Cell Transplantation] explode all trees (222)

#4. (Peripheral Blood Stem Cell Transplantation or Peripheral Stem Cell Transplantation or Stem Cell Transplantation, Peripheral or Transplantation, Peripheral Stem Cell):ti,ab,kw (2,354)

#5. MeSH descriptor: [Bone Marrow Transplantation] explode all trees (1,550)

#6. (Bone Marrow Transplantation or Grafting, Bone Marrow or Bone Marrow Grafting or Transplantation, Bone Marrow or Bone Marrow Cell Transplantation or Transplantation, Bone Marrow Cell):ti,ab,kw (5,761)

#7. MeSH descriptor: [Anemia, Aplastic] explode all trees (237)

#8. (Anemia, Aplastic or Aplastic Anemias or Aplastic Anemia or Aplastic Anaemia or Anaemia, Aplastic or Aplastic Anaemias or Anemia, Hypoplastic or Hypoplastic Anemia or Hypoplastic Anemias):ti,ab,kw (652)

#9. (#1 or #2) and (#7 or #8) (108)

#10. (#3 or #4) and (#7 or #8) (30)

#11. (#5 or #6) and (#7 or #8) (156)
